# Comparative genomics hints at dispensability of multiple essential genes in two *Escherichia coli* L-form strains

**DOI:** 10.1042/BSR20231227

**Published:** 2023-10-25

**Authors:** Yunfei Liu, Yueyue Zhang, Chen Kang, Di Tian, Hui Lu, Boying Xu, Yang Xia, Akiko Kashiwagi, Martin Westermann, Christian Hoischen, Jian Xu, Tetsuya Yomo

**Affiliations:** 1 Laboratory of Biology and Information Science, School of Life Sciences, East China Normal University, Shanghai 200062, PR China; 2 School of Software Engineering, East China Normal University, Shanghai 200062, PR China; 3 Tongji University Cancer Center, Shanghai Tenth People’s Hospital, School of Medicine, Tongji University, Shanghai 200072, China; 4 Faculty of Agriculture and Life Science, Hirosaki University, Hirosaki 036-8561, Japan; 5 Center for Electron Microscopy, Medical Faculty, Friedrich–Schiller–University Jena, Ziegelmühlenweg 1, D-07743 Jena, Germany; 6 CF Imaging, Leibniz Institute On Aging, Fritz–Lipmann–Institute (FLI), Beutenbergstraße 11, 07745 Jena, Germany

**Keywords:** Bacterial cell wall, Essential genes, Genome sequence, L-form, Mutation

## Abstract

Despite the critical role of bacterial cell walls in maintaining cell shapes, certain environmental stressors can induce the transition of many bacterial species into a wall-deficient state called L-form. Long-term induced *Escherichia coli* L-forms lose their rod shape and usually hold significant mutations that affect cell division and growth. Besides this, the genetic background of L-form bacteria is still poorly understood. In the present study, the genomes of two stable L-form strains of *E. coli* (NC-7 and LWF+) were sequenced and their gene mutation status was determined and compared with their parental strains. Comparative genomic analysis between two L-forms reveals both unique adaptions and common mutated genes, many of which belong to essential gene categories not involved in cell wall biosynthesis, indicating that L-form genetic adaptation impacts crucial metabolic pathways. Missense variants from L-forms and Lenski’s long-term evolution experiment (LTEE) were analyzed in parallel using an optimized DeepSequence pipeline to investigate predicted mutation effects (*α*) on protein functions. We report that the two L-form strains analyzed display a frequency of 6–10% (0% for LTEE) in mutated essential genes where the missense variants have substantial impact on protein functions (*α*<0.5). This indicates the emergence of different survival strategies in L-forms through changes in essential genes during adaptions to cell wall deficiency. Collectively, our results shed light on the detailed genetic background of two *E. coli* L-forms and pave the way for further investigations of the gene functions in L-form bacterial models.

## Introduction

Determining essential genes is crucial for understanding the growth and survival mechanisms of bacteria, as well as for developing new drugs and therapies and uncovering minimal gene sets required for life. To date, high-throughput screening methods, comparative genomics, and functional analysis with both *in silico* and *in vitro* approaches have been employed to study essential gene sets and investigate their conservation, functions, and interactions in various bacterial species [1–13]. Most of the essential genes identified are involved in critical cellular processes, genetic information processing, and fundamental metabolisms, some of which are conserved across bacterial species [14]. However, the comparison of experimentally determined essential gene sets across different bacterial species has also shown disparities, indicating that essential gene sets vary between species [12,13,15]. Growing evidence revealed that the definitions of gene essentiality or minimal gene sets are associated with growth conditions, genomic context, horizontal gene transfer, and other potential factors [16,17].

An example of discrepancies in essential genes is the FtsZ (Z-ring)-based cell division machinery in cell wall-deficient (L-form) bacterial models [18,19]. Unlike the Z-ring-based binary fission in modern bacteria, L-forms of various species, either Gram-positive or -negative (such as *Bacillus subtilis*, *Escherichia coli*, *Staphylococcus aureus*, *Corynebacterium glutamicum*, and* Listeria monocytogenes*) divide following spontaneous biophysical changes in cell shape and volumes like the division mechanism of giant lipid vesicles, for example, extracellular blebbing, intracellular budding, and extrusion-resolution [18–24]. Remarkably, these characteristics are similar to that in *Mycoplasma mycoides* with the synthesized minimal bacterial genome JCVI-syn3.0, lacking the critical cytoskeletal components of FtsZ [14,25,26].

From the 1970s, some stable L-form cells from *E. coli*, including LWF+ and NC-7 strains, have been reported, investigating their morphologies, cellular components, and genetics [27–31]. These two stable L-forms were induced under different conditions described previously [27,28]. Besides the common β-lactam inducer Penicillin G for LWF+, lysozyme (peptidoglycan *N*-acetylmuramoylhydrolase) and mutagen *N*-Methyl-*N*′-nitro-*N*-nitrosoguanidine (MNNG) have been used in adapting the NC-7 strain [28]. Long-time exposure to such inhibitor-containing medium forces bacterial cells to accumulate mutations during the L-form transitions [32,33]. Currently, the partial analysis of the *dcw* (division and cell wall) cluster for LWF+ and the whole genome sequence of NC-7 have been described [30,31]. However, detailed genetic information on the variability between these two stable *E. coli* L-form strains is yet to be closely examined. Specifically, genes previously validated as essential for *E. coli* in nutrient-rich media have not yet been investigated in an L-form background [9,34].

In the present study, we determined the whole genome sequences of two previously established *E. coli* L-forms and performed comparative genomic analysis using the verified gene variants during adaptive evolution. We revealed unique and overlapping genes and the affected essential pathways in each L-form strain. The results from the missense variant effect predictions suggest that 6–10% of mutated essential genes are highly deleterious, indicating that multiple essential genes in bacterial L-forms are dispensable. Together, based on the genetic background of existing *E. coli* L-form bacteria, our results are helpful for the further characterization of mutated genes in the L-form bacterial model, paving the way for the definition of a minimal genome and their use in synthetic cell projects.

## Materials and methods

### Bacterial strains

*E. coli* K-12 strain MG1655 was maintained in our laboratory. The stable L-form *E. coli* LWF+ (LW1655F^+^) derived from *E. coli* K12 W1655 F^+^ was donated by Dr Christian Hoischen (Fritz–Lipmann–Institute, Germany) [31,35]. Another stable *E. coli* L-form NC-7 derived from *E. coli* K12 3301 was originally obtained by Onoda et al. [36] and was gifted by Dr Akinobu Oshima (Shimane University, Japan).

### Culture conditions

*E. coli* MG1655 was grown in Luria-Bertani (LB) broth (1% tryptone, 0.5% Yeast Extract, 170 mM [1%] NaCl). Following ingredients from the literature, we successfully cultured both L-form strains in either solid or liquid medium [27,28]. In the present study, LWF+ was cultured with shaking in brain heart infusion (BHI) broth (100 U/ml PenG) without supplementing horse serum, yeast extract, and osmotic stabilizer [31]. In the case of NC-7, the osmoprotective medium MLB medium containing 340 mM NaCl (1% peptone, 0.5% Yeast Extract, 30 mM glucose, 340 mM NaCl, 1 mM CaCl_2_, 25 mM MOPS pH 7.0, 100 U/ml PenG) was used [30] for static culture without shaking. It should be noted that both stable L-forms can grow without the addition of PenG in their culture medium. All bacteria except NC-7 were incubated at 37°C with shaking (200 rpm) while NC-7 cells were incubated at 30°C statically without shaking.

### Genome DNA sequencing and bioinformatic analysis

Whole-genome sequencing was carried out using the Illumina NovaSeq 6000 system at Personalbio Technology Company (Shanghai, China). Genomic DNA was extracted using the Magen Bacterial DNA KF Kit (Sangon, Shanghai, China), and gDNA libraries of each L-forms (<10 passages of the original strains) were constructed by TruSeq DNA PCR-free prep kit (Illumina, San Diego, CA, U.S.A.). Raw reads (each ∼150 bps, BioProject accession number PRJNA905352) were processed to evaluate the quality by FastQC v.0.11.9 (http://www.bioinformatics.babraham.ac.uk/projects/fastqc), trim low-quality reads and remove Illumina adapter sequences using Trimmomatic v.0.39 [37]. According to the GATK Best Practices Workflow, filtered reads were assembled to the reference genome (*E. coli* str. K-12 substr. W3110 (NC_007779.1) for NC-7, *E. coli* str. K-12 substr. MG1655 (NC_000913.3) for LWF+) by bwa v.0.7.17 [38]. It is noteworthy that LWF+ is an *E. coli* K-12 derivative and the genome of K-12 MG1655F- (U00096, identical with NC_000913.3) was used as the reference to analyze the *dcw* cluster [31]. The alignment was sorted by SAMtools v.1.15.1 [39,40] and duplicated reads were removed with the MarkDuplicates tools in GATK v.4.2.2 [41]. Single-nucleotide polymorphisms (SNPs) and insertions/deletions (InDels) were called by HaplotypeCaller (SNP filtration: QD < 2.0 || MQ < 40.0 || FS > 60.0 || SOR > 3.0 || MQRankSum < -12.5 || ReadPosRankSum < -8.0; Indel filtration: QD < 2.0 || FS > 200.0 || SOR > 10.0 || MQRankSum < -12.5 || ReadPosRankSum < -8.0) in GATK v.4.2.2 [41,42], and SnpEff v.5.1 was used to annotate variants and predict the effects [43]. The InDels were verified in IGV (Integrative Genomics Viewer) v.2.12.3 [44] and some false positive variants were removed by CleanSeq [45]. Finally, the genes with missense variants and InDels were selected for follow-up analysis. The gene ontology (GO) and Kyoto Encyclopedia of Genes and Genomes (KEGG) pathway enrichment analysis were performed by clusterProfiler v.4.2.2 [46]. The association of genes was analyzed and visualized in STRING (Search Tool for the Retrieval of Interacting Genes/Proteins) v.11.5 [47]. The information of gene essentiality of *E. coli* was adopted according to the previous literature and database [9,34,48].

### Predicting the functional effect of missense variant

A deep-learning-based model was used to predict the effects of missense variants [49]. After obtaining the missense variants, we followed the guidelines of DeepSequnce pipeline to predict the variant effect scores (*α*) with an optimized deep-learning model according to the self-attention mechanism reported previously [50,51]. Detailed code used for the present study is publicly available at GitHub (https://github.com/kch4221/Deep_missense_variant). The protein domain information for each missense variant was adopted from the Pfam database [52]. Then the domain sequences were loaded to jackhammer (http://www.hmmer.org) to get multi-sequence alignments which were treated as the training dataset. A variational autoencoder with self-attention layers was employed to learn the features of the cluster of sequences. Scaled ΔELBO (evidence lower bound) of wild-type sequences and the mutated sequences generated by the model were used to evaluate the variant effect scores (*α*). Briefly, the normalized variant effect scores (*α*) for a specific SNP have three outcomes: (i) *α*>1 (enhanced; variants hold more activity than wild-type), (ii) *α*<1 (depressed; variants hold less activity than wild-type), and (iii) *α*=1 (unaffected, variants hold wild-type activity). In the present study, we consider a high possibility of loss-function mutations where *α*<0.5 (*α*<0.5: high; 0.5<*α*<0.8: medium; 1.0<*α*>0.8: low). The distributions for all the obtained scores (all genes or essential genes) from each strain were simulated by using Kernel Density Estimation (KDE) method.

## Results

### Gene mutations in L-form strains

**Figure 1:**
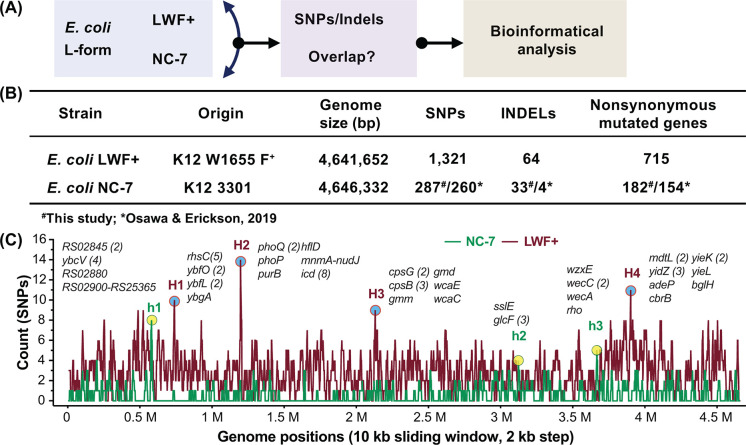
Basic information for two stable *E. coli* L-forms: LWF+ and NC-7 (**A**) The content and experimental flow in this study. The sequencing data of the two L-form strains were analyzed separately, and then the possible overlapped mutant genes were compared and analyzed for functions. The general information from assembled raw reads and mutations (SNPs or INDELs; Nonsynonymous sites) of two L-form strains are summarized in (**B**). The results of NC-7 from the present study (#) were also compared with a previous one [30] indicated by an asterisk (*). All the mutations were presented along with the reference genome used for SNP analysis, with several possible top hotspots flagged (**C**). The genes or intergenic regions were marked according to the number of mutation sites (SNPs, either synonymous or nonsynonymous substitutions) indicated. A 10 kb sliding window with a 2 kb step was employed to screen and visualize the likely targeted regions for each stable L-forms. The potential hotspots for genome mutations are indicated by circles, H1-4 (blue, LWF+) and h1-4 (yellow, NC-7), respectively. The mutated genes were also listed with the number of mutations: *gene name* (*n*). For example, there are five mutations in *rhsC* gene in the hotspot region H1 (0.73–0.74 Mb) of LWF+.

To gain and compare complete genetic information of the two available L-forms, we performed whole genome sequencing of LWF+ and NC-7 followed by detailed bioinformatic analyses (Figure 1A). As summarized in Figure 1B, LWF+ has 1321 SNPs and 64 InDels mutations and 715 genes are still affected by nonsynonymous mutations after filtering out many synonymous mutations listed in Supplementary Table S1. Comparatively, NC-7 has significantly fewer mutation sites than LWF+, that is, 287 SNPs and 33 InDels, and 182 genes hold nonsynonymous mutations (Supplementary Table S1). Although the mutant positions for NC-7 overlapped substantially with those reported by a previous study [30], an increase in the number of both SNP and InDel (Figure 1B) variants was observed. To filter out false positive mutations detected during variant calling, we manually confirmed the existence of those additional SNPs and InDels via the visualization software IGV and CleanSeq, as we established previously [45]. The same trend has also been found in the LWF+ strain compared with the partial sequencing results on the *dcw* gene cluster [31]. The same mutations were verified in *mraY* (Gly295fs, ∆62 aa), *ftsW* (Asp85Tyr), *murG* (Ile119Val), *ftsQ* (Trp132*), and *ftsA* (Ala116Glu), whereas new mutations are found in *mraY* (Met321Thr) and *murC* (Asn216His), and no mutation is detected in *ftsZ*. The discrepancy may arise from mutations in several DNA repair-associated genes (*mutator*, e.g*.*, *mutT* and *mug* in NC-7, *mutM*, *mutS*, and *mutY* in LWF+), resulting in loss of stability in the two L-form genomes and a higher frequency and accumulation of mutations during bacterial culture and passage [30,31,53,54]. To investigate if there are any hotspots for the accumulated mutations, we analyzed the genomic distribution of SNP mutations in two L-form strains by a 10 kb sliding window with a 2 kb step. As demonstrated in Figure 1C, several putative hotspots for mutations were identified, including 0.73–0.74 Mb (H1), 1.188–1.198 (H2) Mb, 2.122–2.132 (H3) Mb, and 3.892–3.902 (H4) Mb for LWF+ and 0.574–0.584 (h1) Mb, 3.116–3.126 (h2) Mb, and 3.66–3.67 (h3) Mb for NC-7. However, we did not observe overlapped regions between potential hotspots from these two L-forms, indicating that most SNPs may emerge during adaption and evolution following continuous culture in the presence of β-lactam, lysozyme, or the mutagen MNNG [27,28].

### Comparative genomic analysis between LWF+ and NC-7 strains

**Figure 2:**
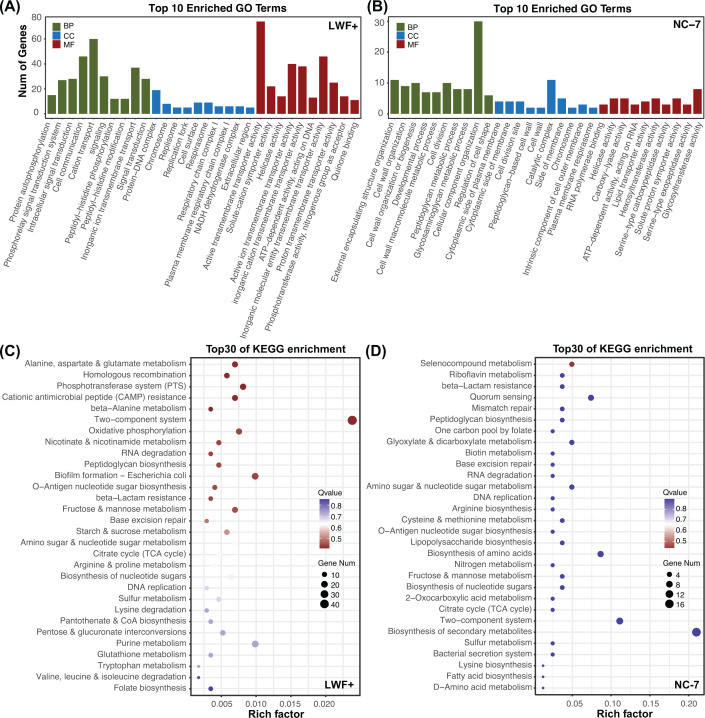
Overview and enrichment analysis of mutated genes from genome re-sequencing of *E. coli* LWF+ and NC-7 The mutated genes were analyzed for enrichment in three GO ontologies: biological process (BP), cellular component (CC), and molecular function (MF). The top 10 enriched GO terms sorted by number were visualized as bar graph in (**A**: LWF+) and (**B**: NC-7). Top 30 KEGG pathways sorted by predicted Q-value for mutated genes in each strain (left panel: LWF+; right panel: NC-7) are summarized in (**C,D**), respectively. Some pathways, such as peptidoglycan biosynthesis and β-lactam resistance predicted to be relevant to the formation and division of these two L-forms, are shown. The number of genes and Q-value in each pathway were shown in closed circles with dark blue to red gradient color. A greater rich factor (number of mutated genes/total number of genes) in the individual pathway indicates a greater degree of enrichment.

GO and KEGG enrichment analyses were performed on the mutated genes to identify the biological functions and pathways that might be affected in each L-form strain. The detailed enrichments from the GO analysis (Top 10 enriched GO terms in number) are further summarized in Figure 2A (LWF+), Figure 2B (NC-7), and Supplementary Table S2. Generally, a greater gene number and enrichment can be seen in LWF+, possibly due to its higher mutation frequency. In NC-7, genes involved in the cell wall, cell division, cell shape, peptidoglycan, membrane, lipid in the biological process (BP) and cellular component (CC) categories are significantly enriched. Some other GO terms from the molecular function (MF) category, such as RNA polymerase binding, helicase activity, carboxy-lyase activity, and ATP-dependent activity, are also likely altered. In the case of LWF+, mutated genes from signal transduction (BP), cell communication (BP), cation transport (BP), protein–DNA complex (CC), respirasome (CC), and cell transmembrane transporter (BP, MF) are enriched. These results are directly reflected in the KEGG enrichment analysis, where the top 30 affected pathways sorted by Q-value are shown in Figure 2C (LWF+), 2D (NC-7), and Supplementary Table S3. In LWF+, besides some L-form-relevant pathways, previously reported for Gram-positive *B. subtilis* [19,55–57], such as oxidative phosphorylation, peptidoglycan biosynthesis, and β-lactam resistance, other unexpected enrichments in some pathways are also observed. These include alanine, aspartate, and glutamate metabolism, homologous recombination, phosphotransferase system (PTS), cationic antimicrobial peptide (CAMP) resistance, two-component system, nicotinate, and nicotinamide metabolism, RNA degradation, and so on (Figure 2C). Similarly, peptidoglycan biosynthesis, β-lactam resistance, and fatty acid biosynthesis are enriched in NC-7 as expected, together with some previously unreported pathways such as selenocompound metabolism, quorum sensing, riboflavin metabolism, and glyoxylate and dicarboxylate metabolism (Figure 2D). Collectively, both *E. coli* L-forms hold several mutations that are not predicted to be directly associated with the formation of L-forms by known pathways like peptidoglycan and lipid biosynthesis [57].

**Figure 3:**
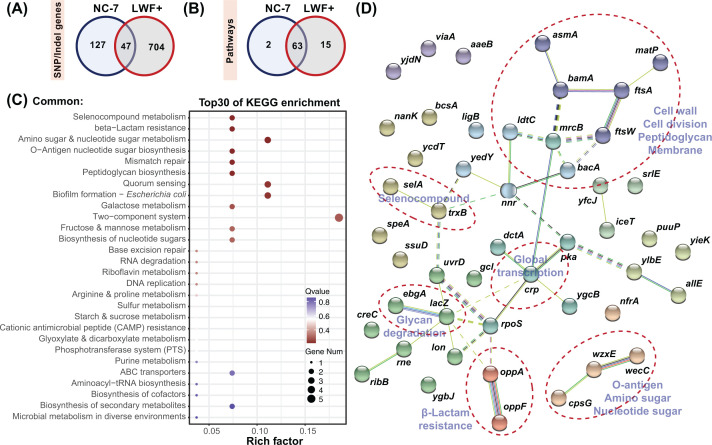
Comparative genomic analysis of mutated genes in two *E. coli* L-forms (**A**) Venn diagram showing the number of mutated genes in predicted KEGG pathways and the ones shared by the two L-form strains. Panel (**B**) shows the same diagram for number and overlap in enriched pathways. The overlapped top 30 KEGG pathways predicted from mutated genes were summarized in (**C**). Critical pathways potentially related to the formation and division of L-forms are in blue. (**D**) Possible network information between overlapping mutated genes was simulated in STRING. The networks consist of the total number of nodes (proteins involved) and number of edges (PPI connections among nodes). The color of the nodes is for visual representation. Enriched gene networks, such as cell wall, cell division, glycan degradation, β-lactam resistance, and global transcription, are circled by red dashes.

We characterized commonalities or specificities in these two *E. coli* L-forms with a comparative analysis (shown in Figure 3). Despite the significant differences in the number of mutations between LWF+ and NC-7, there are 47 common genes and 63 overlapped metabolic pathways (Figure 3A), although mutation positions on common genes are different (Supplementary Tables S4 and Table S5), indicating some genes or pathways (e.g., SEDS family genes which are responded for cell septation, elongation, division, and sporulation) might be specifically targeted in the process of L-form induction and adaption as demonstrated in MutationDB [58].

The KEGG enrichment analysis for 47 common mutant genes shows that selenocompound metabolism (*trxB*/*selA*), β-lactam resistance (*oppA*/*oppF*), amino sugar and nucleotide sugar metabolism (*cpsG*/*nanK*/*wecC*), O-Antigen nucleotide sugar biosynthesis (*cpsG*/*wecC*), mismatch repair (*ligB*/*uvrD*), peptidoglycan biosynthesis (*mrcB*/*bacA*), quorum sensing (*oppA*/*oppF*/*crp*), biofilm formation (*rpoS*/*crp*/*bcsA*), galactose metabolism (*lacZ*/*ebgA*), two-component system (*mdtD*/*crp*/*dctA*/*wecC*/*creC*), etc. (Figure 3C and Supplementary Table S6) are involved. Two KEGG pathways are enriched in both L-forms, including β-lactam resistance and peptidoglycan biosynthesis, which are closely related to L-form adaptions. Similarly, we also performed protein interaction analysis for these 47 overlapped mutant genes as illustrated in Figure 3D. Mutant genes related to (i) the cell wall, cell division, peptidoglycan, membrane (e.g*.*, *ftsW*, *ftsA*, *mrcB*, *bacA*), (ii) O-antigen (e.g*.*, *wzxE*, *wecC*), and (iii) β-lactam resistance (e.g., *oppA*, *oppF*), and other pathways such as global transcription do interact with each other, either directly or indirectly, which corroborates the results of KEGG enrichment analysis. Taken together, these results show that the L-forms might require other beneficial mutations for proliferation or survival during evolutionary adaption, such as cAMP-activated global transcriptional regulator (*crp*) to adjust global cellular transcription [59,60].

### Essential gene mutations in L-forms

Frameshift mutations caused by insertions or deletions of DNA fragments may have a great impact on protein functions. On the other hand, the impact of missense SNP mutations is difficult to assess and need detailed experimental verification to confirm the altered functionality of mutant proteins. It is challenging to draw convincing conclusions for the large number of SNPs found in both L-forms, especially those identified in essential genes for *E. coli* (Supplementary Table S1). The validation of protein function for hundreds of variants is experimentally unfeasible. Therefore, we deployed an optimized deep-learning model to quantitatively simulate the missense variant effect based on the available pipeline *DeepSequence* [49].

**Figure 4:**
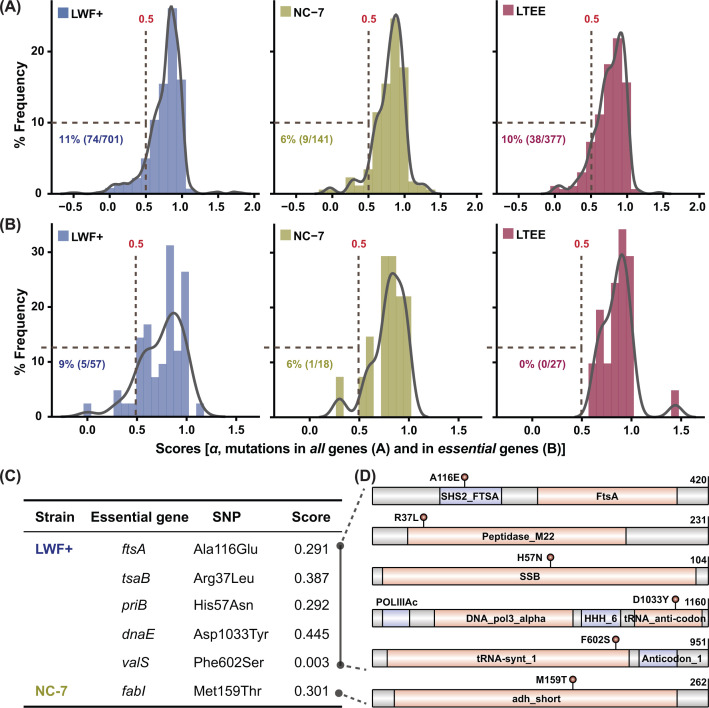
Predicted missense variant effects in mutations in all genes by an optimized deep-learning method The missense mutation sites were further analyzed using a deep mutational scanning approach to estimate scores (*α*, *x*-axis) to indicate the functional effect of missense variants. Sequencing results from long-term experimental evolution (LTEE) were also adopted from Dr Lenski’s previous publications [61], and compared with L-form strains. The frequency distribution (*y*-axis) of mutations in all genes (**A**) or essential genes (**B**) from LWF+ (blue), NC-7 (green), and LTEE (red), were plotted and compared. A predicted score for a mutation site lower than 0.5 (*α*<0.5, gray dashed lines) was considered to affect protein function significantly. Six essential genes (*ftsA*, *tsaB*, *priB*, *dnaE*, and *valS* from LWF+, *fabI* from NC-7) with mutations with a predicted score below 0.5 (*α*<0.5) were listed in (**C**). The detailed mutation sites in six essential genes with predicted functional domains were shown in (**D**).

As a result, we obtained all variants’ effect scores for both L-forms (141 SNPs for NC-7 and 700 SNPs for LWF+, Table S7), and plotted frequency distributions for scores ranging from -0.6 to 2.0, as shown in Figure 4A. Based on the prediction results, most variants’ *α* scores lie within the range of 0.5–1.0, suggesting that those missense mutations exert only a weak or negligible effect on protein function. This conclusion is consistent with the distribution of the variant effect scores for 377 gene mutations (40K generations) from Lenski’s *E. coli* long-term evolutionary experiment (LTEE), where *E. coli* cells were cultured with minimal medium supplemented with glucose as the only carbon nutrient [61]. Figure 4A shows the probability density estimates by KDE of variant effect scores for all mutated genes in *E. coli* L-forms and LTEE strains, with the highest frequency of occurrence being around *α*=0.8. After evolutionary adaptions, L-forms and LTEE showed approximately 6–11% SNPs (6% for NC-7, 11% for LWF+, and 10% for LTEE) that might negatively affect the protein functions (*α*<0.5, listed in Supplementary Table S7). When we focused only on mutations in previously reported essential genes [9,34], we found that only the two L-form strains hold mutations with low variant effect scores (*α*<0.5, Supplementary Table S8), which is consistent with the enhanced function mentioned in Lenski’s findings [61]. This result indicates that certain biological networks other than PG and membrane synthesis are subjected to adaption under cell wall-targeted environments, which may differ dramatically from those in walled cells. Mutations in five genes (*ftsA*, *tsaB*, *priB*, *dnaE*, and *valS*) have low *α* score predictions in LWF+, while one gene (*fabI*) has *α*<0.5 in NC-7 (Figure 4C). The five essential proteins in LWF+ are involved in cell division (*ftsA*, [62]), DNA replication (*priB*, *dnaE*, [63,64]), and tRNA synthesis (*tsaB*, *valS*, [65,66]), whereas *fabI* from NC-7 is responsible for fatty acid synthesis [67]. All SNPs are marked into each essential gene in Figure 4D, showing that these SNPs are located in the functional domain of each protein.

## Discussion

We sequenced the genome of two stable *E. coli* L-form strains, revealing frequent genetic mutations status across the entire genome (Figure 1). Unsurprisingly, both L-form strains share mutated genes related to cell wall biosynthesis and β-lactam resistance. However, we also report mutations in crucial genes, suggesting that essential metabolic pathways in L-forms could be affected (Figures 2 and 3). Despite these complex genetic alterations, the adapted *E. coli* L-forms could survive and proliferate. Our results indicate that L-forms undergo changes in gene essentiality during induction and/or long-term adaption to their environment, with stark differences from classic LTEE (Figure 4). Further investigation in what this change may entail are needed to elucidate the functional alteration for mutated essential genes in L-form background.

Through whole genome sequencing, cell wall biosynthesis-related pathways were identified as targets in both L-forms. We discovered eight mutated genes in LWF+ are involved in the peptidoglycan biosynthesis, including *murC*, *mraY*, *murG*, *bacA*, *mrcA*, *mrcB*, *dacA*, and *dacB*. The *mraY* gene codes the enzyme that catalyzes the synthesis of the first lipid intermediate of peptidoglycan (Table 1). Cell division is prevented in the *mraY* deficient mutant, eventually leading to cell death [68]. The frameshift mutation caused by the base deletion produces a loss-of-function MraY protein lacking the C-terminal 62 amino acids (∆62 aa) in *E. coli* LWF+, which might be one of the key mutations leading to reduced cell wall synthesis. The MurG protein is essential for the final intracellular step of peptidoglycan subunit assembly [69] and has a isoleucine-to-valine amino acid point mutation at 119 (Ile119Val) in LWF+, but it is unclear if this amino acid substitution affects protein functions (*α*=0.993, low effect). Mutations in the *mraY* and *murG* genes are consistent with the results obtained by Siddiqui et al. for sequencing the *dcw* gene cluster [31]. Another single base mutation in the essential *murC* gene (His216Asp) was highlighted by our genomic sequencing. It is known that the MurC protein adds the first amino acid of the peptide moiety in the assembly of the monomer unit of peptidoglycan [70]. This might be a new mutation that emerged during recent transmission culture. The effects of this missense mutation remain to be studied (*α*=0.610, medium effect). Except for the three genes within the *dcw* gene cluster, the remaining five genes are not lethal when altered in isolation [71–75]. Notably, cells lacking *mrcA* or *mrcB* alone do not exhibit growth or cell morphology defects, while a *mrcA*/*mrcB* double mutation is lethal [74]. In the penicillin-binding protein 1a (PBP1A, *mrcA*) and PBP1B (*mrcB*) proteins, we coincidentally discover substitutions at aa761 (Asp761Tyr, *α*= 0.618, medium effect) and 656 (Pro656Leu, *α*=0.707, medium effect), respectively. The impact of these two simultaneous mutations on the L-forms may be an intriguing area to explore, since it might be directly targeted by PenG. By binding to penicillin-binding proteins (PBPs), β-lactam antibiotics hinder bacterial cell wall synthesis, which leads to cell death [76]. Furthermore, the additional six genes from LWF+ involved in the β-lactam resistance pathway hold missense mutations, which could be responsible for its ability to grow against β-lactam antibiotics. The six genes include *ampG*, whose protein product regulates β-lactamase expression; *acrB*, which encodes a component of the drug efflux pump; *mrcA*, which encodes a penicillin-binding protein; and *oppA*/*oppC*/*oppF*, which encodes components of the oligopeptide (Opp) transport system [77–81].

**Table 1:** Partial key mutated genes discovered in LWF+ and NC-7 L-forms

L-form	Gene	Essentiality	Mutations	Score (*α*)	Impact	Annotations
LWF+	*ftsA*	Yes	Ala116Glu	0.291	High	ATP-binding cell division FtsK recruitment protein
	*mraY*	Yes	Gly295*fs*	-	High	Phospho-N-acetylmuramoyl-pentapeptide transferase
	*murC*	Yes	Asn216His	0.610	Medium	UDP-N-acetylmuramate:L-alanine ligase
	*murG*	Yes	Ile119Val	0.993	Low	N-acetylglucosaminyl transferase
	*bacA*	No	Thr47Ala	0.824	Low	Undecaprenyl pyrophosphate phosphatase
	*mrcA*	No	Asp761Tyr	0.618	Medium	Murein transglycosylase and transpeptidase
	*mrcB*	No	Pro656Leu	0.707	Medium	Fused glycosyl transferase and transpeptidase
	*dacA*	No	Gly288Asp	0.592	Medium	D-alanyl-D-alanine carboxypeptidase
	*dacB*	No	Gln55Lys	0.925	Low	D-alanyl-D-alanine carboxypeptidase
			Glu218Lys	0.793	Medium	
	*mutY*	No	Ser215Arg	0.864	Low	Adenine glycosylase active on G-A mispairs
	*mutM*	No	Gly241Ser	0.665	Medium	Formamidopyrimidine/5-formyluracil/5-hydroxymethyluracil DNA glycosylase
	*uvrD*	No	Tyr230Phe	0.456	High	DNA-dependent ATPase I and helicase II
			Ala715Val	0.998	Low	
	*ligB*	No	Gly59Asp	0.803	Low	DNA ligase
NC-7	*ftsA*	Yes	Pro361Ser	0.744	Medium	ATP-binding cell division FtsK recruitment protein
	*ftsI*	Yes	Leu393*fs*	-	High	Transpeptidase involved in septal peptidoglycan synthesis
	*mrcB*	No	Pro790Ser	0.889	Low	Fused glycosyl transferase and transpeptidase
	*bacA*	No	Ser27Arg	0.390	High	Undecaprenyl pyrophosphate phosphatase
	*fabI*	Yes	Met159Thr	0.301	High	Enoyl-[acyl-carrier-protein] reductase
	*mutT*	No	Pro117Ser	0.894	Low	dGTP-preferring nucleoside triphosphate pyrophosphohydrolase
	*mutS*	No	Arg455_Glu456*ins*GluArg	-	Medium	Methyl-directed mismatch repair protein
	*uvrD*	No	Ala142Val	0.867	Low	DNA-dependent ATPase I and helicase II
	*ligB*	No	Val130Ile	0.731	Medium	DNA ligase
	*Mug*	No	Glu84Lys	0.748	Medium	G/U mismatch-specific DNA glycosylase

*fs*: frameshift; *ins*: insertion.

Similar to LWF+ (*murC*, *mraY*, *murG*, *bacA*, *mrcA*, *mrcB*, *dacA*, and *dacB*), mutations in NC-7 also involved the peptidoglycan biosynthesis and β-lactam resistance pathways, and the mutant genes in the two pathways were *ftsI* (Leu393fs, frameshift, high effect)/*mrcB* (Pro790Ser, *α*=0.889, low effect)/*bacA* (Ser27Arg, *α*=0.390, high effect), and *ftsI* (Leu393fs, frameshift, high effect)/*oppA* (Asn271Tyr, *α*=1.144, enhancement effect)/*oppF* (Ser325Ala, *α*=1.251, enhancement effect), respectively. The essential *ftsI* gene (PBP3 protein), a target of β-lactam antibiotics, is a central component of the divisome in *E. coli*, catalyzing cross-linking of the cell wall peptidoglycan during cell division [82]. Our sequencing results revealed a frameshift mutation occurred after aa393 of PBP3 protein in NC-7. According to the crystal structure of the PBP3 protein, 4 of the 8 amino acid residues necessary for the transpeptidase activity and binding to β-lactam antibiotics come after aa393 [83]. Therefore, mutations in the *ftsI* gene in NC-7 may be the leading cause of the absence of cell walls and resistance to β-lactams. Interestingly, *mrcB* and *bacA* are mutated in both *E. coli* L-forms (Table 1 and Supplementary Table S4). Since the residues 781–844 of PBP1b (*mrcB*) are dispensable, the substitution of Pro790Ser in NC-7 should have a low impact on its function [84,85]. In LWF+, Pro656Leu (*α*=0.707, medium effect) of PBP1b and Thr47Ala (*α*=0.824, low effect) of BacA was also spotted, implying *mrcB* and *bacA* might be targeted and important in PenG-induced L-forms. The mutation of BacA occurred at the aa27 (Ser27Arg, *α*=0.390, high effect) with high impacts according to a previous functional analysis that a Ser27Ala mutant resulted in an almost total loss of phosphatase activity [86]. It is worth noting that the mutations of OppA (Asn271Tyr) and OppF (Ser325Ala) in LWF+ and NC-7 are accidentally identical, while the mutation site did not fall into a known functional position [81,87,88]. However, it is extremely rare for the mutation sites to be identical, proposing that *oppA* and *oppF* possibly play a critical role in L-form formation and growth. Previous research has demonstrated that excessive fatty acid synthesis in L-forms can result either directly by overexpression of fatty acid synthesis genes or indirectly by lack of cell wall production [19,57]. The missense mutation in *fabI*, an essential gene for the fatty acid synthesis process, has also occurred (Supplementary Table S1) at Met159Thr (*α*=0.301, high effect), which is a triclosan binding site, and mutations in this domain could result in Triclosan resistance [89].

In addition to cell wall-related pathways, certain auxiliary gene mutations may be necessary for the growth of the two L-forms used in the present study. The previous findings in the *B. subtilis* LR2 L-form model revealed that inhibiting the synthesis of cell walls caused an abnormal rise in reactive oxygen species (ROS), preventing L-form cell growth. However, mutations that counteract ROS, adding exogenous ROS scavengers, or anaerobic culture conditions, can promote the growth of various L-form cells, including *E. coli* [90]. Four of the five redox chain complexes in the LWF+ contain gene mutations: *nuoL* (Ala41Thr, *α*=0.842, low effect), *nuoH* (Pro267Ser, *α*=0.978, low effect), *nuoG* (His427Arg, *α*=0.268, high effect), *nuoF* (Glu106Asp, *α*=0.696, medium effect), and* nuoC* (Asn397Ser, *α*=0.863, medium effect), which encode the NADH dehydrogenase I components [91]; *sdhA* (Lys314Glu, *α*=0.666, medium effect) and *sdhD* (Tyr29Leu, *α*=0.774, medium effect) encoding two catalytic subunits in the four subunit succinate dehydrogenase (SQR) enzyme [92,93]; *cyoE* (Ala29Thr, *α*=0.785, medium effect), *cyoB* (Gly28fs, frameshift, high effect), and *cyoA* (Ala249Thr, *α*= 1.051, low effect) involved in the compositions and catalytic function of the cytochrome bo(3) oxidase complex, where the *cyoB*-encoded protein is frameshifted at aa28 and loses quinol oxidase activity [94]; and *ppk* (Arg132His, *α*=0.679, medium effect), *atpB* (Gly173Asp, *α*=0.814, low effect), and *atpH* (Leu133Met, *α*=0.649, medium effect) involved in the function of ATP synthase [95,96]. Besides, up to 13 genes (Supplementary Table S2) in the oxidative phosphorylation pathway have mutations, indicating that the redox process may be closely related to L-form’s growth. In addition to these pathways, mutated genes in the base excision repair pathway are also worthy of attention. Oxidative DNA damage is an important factor leading to base mismatches. Guanine in DNA strands is attacked by ROS, leading to the production of 7,8-dihydro-8-oxoguanine (8-oxoG), a base analog with ambiguous base-pairing characteristics that can pair with either A or C during DNA synthesis and reverse the direction of G:C to T:A [97]. In *E. coli*, the repair of 8-oxoG is carried out by the three glycosylases: MutT, MutY, and MutM. MutT hydrolyzes 8-oxo-dGTP alone to 8-oxo-dGMP, preventing 8-oxoG from entering the genome during DNA replication [98]; MutY excises A mismatched with 8-oxoG to decrease the propagation of mistakes [99]; and MutM excises DNA 8-oxoG in the chain [100]. Both the *mutY* and *mutM* genes had missense mutations in the LWF+ genome (Table 1). Studies have revealed that *mutY* and *mutM* double mutants exhibit spontaneous mutation rates that are 25–75 times higher than those of the wild type, and G:C to T:A transversions account for all the base substitutions [101]. Correspondingly, the proportion of G and C mutations in LWF+ was significantly higher than that of A and T mutations (Supplementary Figure S1). However, the ratio of G:C to A:T is slightly higher than that of G:C to T:A, which we speculate is caused by a combination of mutations in other base mismatch genes (*tag*/*ligB*/*polA*). Besides most variants included in a previous report [30], we found hundreds of additional mutations in this round of genome sequencing for NC-7. We noticed that mutations in the genes involved in mismatch repair and base excision repair as *mutators*, including *mutT*/*mutS*/*uvrD*/*ligB*/*mug* (Table 1), might be responsible for generating and accumulating new mutations in NC-7 [53,54,102–108].

Unlike the L-forms induced by modern genetic manipulation approaches for several key genes, both L-form strains here examined hold heavy mutations in their genomes after long-time adaption to cell wall-targeting antibiotics [57]. Given the large number of SNPs in both strains, experimental verification of the effect of each SNP is unfeasible due to the large volume of experiments needed. Thus, we employed a bioinformatic tool as a beneficial filter to systemically analyze the missense effect on most genes found in L-form genomes [49]. As shown in Figure 4, it demonstrated a significant ratio (6–10%) of mutated essential genes that might be dysfunctional (*α*<0.5) in L-forms. We successfully screened out several key factors (*ftsA*, *tsaB*, *priB*, *dnaE*, *valS*, and *fabI*) with higher impact for subsequent evaluations, all of which have been previously identified as essential genes in nutrient-rich medium [9]. The context-dependence of gene essentiality has been extensively discussed, taking into account its variation during evolutionary adaptation and its strain-dependence [12]. Bacterial L-forms could serve as a useful model to study and reconsider the gene essentiality (like the genes listed in Figure 4C) when cell wall synthesis and cell division machinery are genetically deficient, similar to conditions for *M. mycoides* holding a synthetic FtsZ-free minimal bacterial genome JCVI-syn3.0 [14,25,26]. Thus, it will be of great interest to comprehensively investigate the gene (e.g*.*, *fts* gene family and cell division-related genes) function and essentiality in L-forms, either naturally induced or genetically manipulated, under different *in vitro* and *in vivo* conditions [24,109–111].

The results of the present study need to be considered and confirmed through experimental evidence in the future. As discussed above, the essentiality of a specific gene is known to be impacted by both growth conditions and genetic background [12,13,15–17]. The essential genes evaluated and filtered for both L-form and LTEE strains may not truly indicate the gene essentiality in each unique genetic background and culture medium. More experimental evidence is required to investigate the essentiality of filtered genes, and their mutations (shown in Figure 4C, e*.g.*, *ftsA* and *fabI*) under all tested strains and growth conditions. Future studies will also be focused on establishing *E. coli* L-forms with well-defined genetic backgrounds using the gene candidates found in this study, aiming for both fundamental and applied research in synthetic biology using L-form bacteria as a model [23,25,112,113].

## Supplementary Material

Supplementary Figure S1

Supplementary Tables S1-S8

## Data Availability

All supporting data are included within the main article and its supplementary files. The full genome re-sequencing raw data are deposited at BioProject with accession number PRJNA905352.

## Abbreviations

8-oxoG: 7,8-dihydro-8-oxoguanine
CAMP: cationic antimicrobial peptide
LTEE: long-term evolutionary experiment
PBP: penicillin-binding protein
PTS: phosphotransferase system
ROS: reactive oxygen species
