# Supplementary material for: Comparative genomics hints at dispensability of multiple essential genes in two Escherichia coli L-form strains
Source: Biosci Rep. 2023 Oct 25;43(10):BSR20231227. doi: 10.1042/BSR20231227 (PMC10600066; doi:10.1042/BSR20231227)
Supplement: Supplementary Figure S1 [file BSR-2023-1227_supp.pdf]

Fig. S1

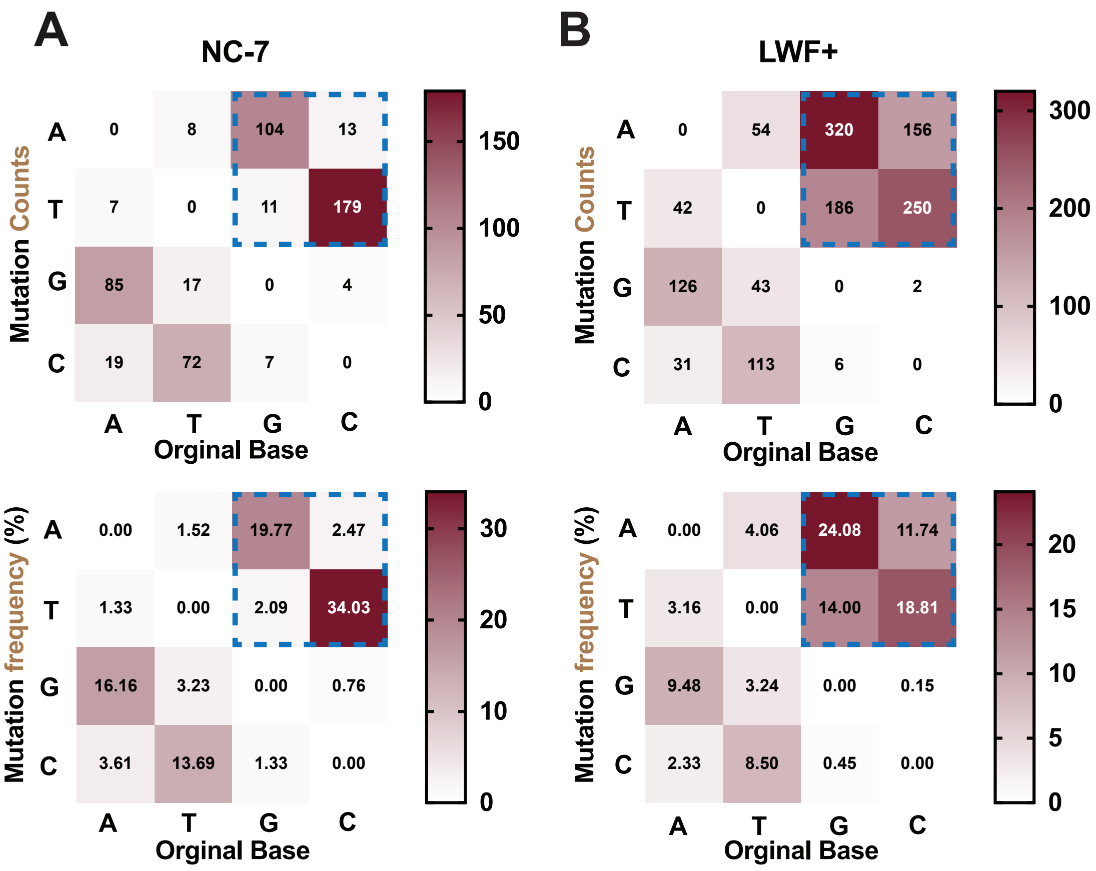

Fig. S1 Analysis of gene mutation trend in the two *E. coli* L-forms, NC-7 (A) and LWF+ (B). Each upper and lower panel shows the mutation counts or frequency alerted from the original base. G->A, C->T in NC-7, and G->A, C->T in LWF+ are indicated as higher mutation species (GC->AT) in each strain, respectively
